# Human monoclonal antibodies protect against viral-mediated pneumococcal superinfection

**DOI:** 10.3389/fimmu.2024.1364622

**Published:** 2024-06-12

**Authors:** Aaron Gingerich, Lauren Mahoney, Anna L. McCormick, Rose J. Miller, Jarrod Mousa

**Affiliations:** ^1^ Center for Vaccines and Immunology, College of Veterinary Medicine, University of Georgia, Athens, GA, United States; ^2^ Department of Infectious Diseases, College of Veterinary Medicine, University of Georgia, Athens, GA, United States; ^3^ Department of Biochemistry and Molecular Biology, Franklin College of Arts and Sciences, University of Georgia, Athens, GA, United States; ^4^ Department of Biomedical Sciences, College of Medicine, Florida State University, Tallahassee, FL, United States

**Keywords:** Streptococcus pnemoniae, RSV (respiratory syncytial virus), hMPV, influenza, monoclonal antibody

## Abstract

**Introduction:**

Community-acquired pneumonia (CAP) is a global health concern, with 25% of cases attributed to *Streptococcus pneumoniae* (*Spn*). Viral infections like influenza A virus (IAV), respiratory syncytial virus (RSV), and human metapneumovirus (hMPV) increase the risk of *Spn*, leading to severe complications due to compromised host immunity.

**Methods:**

We evaluated the efficacy of an anti-PhtD monoclonal antibody (mAb) cocktail therapy (PhtD3 + 7) in improving survival rates in three viral/bacterial coinfection models: IAV/*Spn*, hMPV/*Spn*, and RSV/*Spn*.

**Results:**

The PhtD3 + 7 mAb cocktail outperformed antiviral mAbs, resulting in prolonged survival. In the IAV/*Spn* model, it reduced bacterial titers in blood and lungs by 2-4 logs. In the hMPV/*Spn* model, PhtD3 + 7 provided greater protection than the hMPV-neutralizing mAb MPV467, significantly reducing bacterial titers. In the RSV/*Spn* model, PhtD3 + 7 offered slightly better protection than the antiviral mAb D25, uniquely decreasing bacterial titers in blood and lungs.

**Discussion:**

Given the threat of antibiotic resistance, our findings highlight the potential of anti-PhtD mAb therapy as an effective option for treating viral and secondary pneumococcal coinfections.

## Introduction

Infectious respiratory diseases continue to pose a significant and daunting threat to global public health. A variety of pathogens are responsible for respiratory illnesses, with *Streptococcus pneumoniae* (*Spn)* being a major contributor. This gram-positive bacterium causes an array of infections such as pneumonia, otitis media, sinusitis, and invasive diseases like bacteremia and meningitis (1). Additionally, respiratory viruses like influenza virus, respiratory syncytial virus (RSV), and human metapneumovirus (hMPV) significantly contribute to the global burden of respiratory infections (2). Bacterial/viral co-infections have been a concerning medical phenomenon since the early 1900s when influenza and pneumonia were recognized as public health concerns (3, 4). *Spn* is notorious for its involvement in co-infections with influenza A virus (IAV), RSV, and hMPV (5–10). When these virally infected individuals are also infected with *Spn*, due to colonization or new infection, the resulting synergy often culminates in more severe and complex disease manifestations than single-pathogen infections (3, 4). Research has shown that co-infections can amplify pathogen virulence, which worsens patient outcomes (3, 11). This lethal synergy occurs through various mechanisms such as the destruction of epithelial tissue (12–16), dysregulated immune function, (17–23) and a modified microbial environment (24–27). Thorough investigation into co-infections is crucial as they can lead to severe clinical manifestations across all age groups, but particularly in elderly individuals, infants, young children, and the immunocompromised (11, 28). Although influenza vaccination has proven to decrease hospitalization rates for pneumonia patients (29), and immunization against *Spn* has greatly reduced the incidence of invasive pneumococcal disease (30, 31), the vaccines for *Spn* and IAV offer protection against single infections however (32, 33), they have shown limited protection against co-infection in mice (34–36). Present treatments for these co-infections consist of antibiotic therapy aimed at *Spn* and antiviral medications targeting viruses. Both approaches demonstrate some protective effects (37–39), however, due to the emergence of drug-resistant pathogens (40) and the declining efficacy of existing treatments, there is a pressing need for innovative approaches in tackling these coinfections. Furthermore, gaining an in-depth understanding of dual-pathogen infection mechanisms may pave the way for developing cutting-edge prevention and management strategies.

In our previous research, we discovered that the protective effect of the human mAb PhtD3 during pneumococcal infections was facilitated by macrophages and the complement system (41). Although this protection was significantly reduced in an IAV/Spn coinfection model, we managed to restore protection using a mAb cocktail of PhtD3 + 7 (41). In this study, we established additional lethal coinfection models for both RSV/Spn and hMPV/Spn, and we aimed to explore whether therapeutically targeting the virus with mAbs could offer protection against secondary pneumococcal infections. Our findings demonstrate that the PhtD3 + 7 mAb cocktail provides protection across all three coinfection models, with noticeably enhanced efficacy compared to antiviral mAbs alone. Our findings highlight the exciting potential of innovative therapeutics in tackling *Spn* coinfections.

## Materials/methods

### Ethics statement

All animal studies performed were in accordance with protocols approved by the Institutional Animal Care and Use Committee of the University of Georgia.

### Bacterial strains and growth conditions

Bacterial colonies were grown on BD Trypticase Soy Agar II with 5% Sheep Blood (BD, Franklin Lakes NJ). Bacteria cultures were grown at 37°C in 5% CO2 in Todd-Hewitt broth (BD, Franklin Lakes NJ) supplemented with 0.5% yeast extract for 12 hrs. Cultures were frozen and stored at -80°C with 10% glycerol until used, and following thawing, cultures were washed twice with PBS before being used in experiments. The numbers of CFUs per milliliter of these stocks were determined by plating a single quick-thawed diluted aliquot on sheep’s blood agar plates. The calculated number of CFUs was subsequently used to make dilutions for experiments from aliquots thawed at later times. In each experiment, the actual number of CFUs administered was determined by plating on blood agar at the time of the assay.

### Antibody generation with CHO and 293 cells

mAbs PhtD3, PhtD7 and Ab6649 were generated as previously described in ExpiCHO cells (41, 42). Briefly, ExpiCHO cells were grown in FreeStyleCHO media supplemented with L-glutamine. For transfections, cells were transferred to ExpiCHO media and transfected using the high-titer protocol according to the manufacturer’s instructions. mAbs were purified from culture supernatants using Protein G columns (Cytiva) as previously described (41, 42). mAbs MPV467 and D25 were expressed by transfecting Expi293 cells with HC/LC plasmids and purified from culture supernatant with a Protein G column (Cytiva) as previously described (43).

### Co-infection studies

To establish the model of pneumococcal co-infection with hMPV or RSV, 5-7 week-old BALB/c mice (Jackson Laboratories) were anesthetized by inhalation of 5% isoflurane and intranasally challenged with 50 µL of 5x10^5^ PFU of hMPV TN/93-32 or with 50 µL of 3-5x10^5^ PFU of RSV A2 in PBS. Previous studies have shown this dose to be non-lethal but viral replication does occur (43), Five days post viral infection, mice were anesthetized by inhalation of 5% isoflurane and intranasally challenged with 40 µL of 10^4^, 10^5^, or 10^6^ CFUs of pneumococcal strain WU2 serotype 3 in PBS. Based on these dosing experiments, we utilized 10^6^ CFUs of Spn for the co-infection studies. On day 3 post viral infection, some mice were intraperitoneally inoculated with 10 mg/kg of mAb MPV467 (hMPV infection) or 10 mg/kg mAb D25 (RSV infection). Two hours prior to bacterial challenge, mice were intraperitoneally inoculated with 15 mg/kg of mAb (PhtD cocktail) or PBS.

For the IAV/Spn model, we utilized doses and timepoints as previously described (41). 5-7 weeks old C57BL/6 mice (Charles River) were anesthetized by inhalation of 5% isoflurane and intranasally challenged with 100 FFU of H1N1 A/California/07/2009 in 40 µL of PBS. After 7 days, mice were anesthetized by inhalation of 5% isoflurane and intranasally challenged with 40 µL of 1x10^4^ CFUs of pneumococcal strain WU2 in PBS. Twenty-four hours post viral infection, some mice were intraperitoneally inoculated with 10 mg/kg of mAb Ab6649. Two hours prior to bacterial challenge, mice were intraperitoneally inoculated with 15 mg/kg of mAb or PBS. In all studies above, mice were weighed and assessed daily and were euthanized when >30% of pre-infection body weight was lost, were nonresponsive to manual stimulation, and/or were exhibiting respiratory distress.

### Lung viral titers

To determine viral titers of hMPV or RSV at the time of bacterial infection, mice were euthanized on day 5 post-viral challenge and lungs were collected and homogenized for virus titration as previously described (44). Briefly, RSV-challenged lung homogenates were plated on HEp-2 cells (Opti-MEM+2% FBS) while hMPV-challenged lung homogenates were plated on LLC-MK2 cells (Opti-MEM + 5 μg/mL trypsin-EDTA and 100 μg/mL CaCl_2_) in 24 well plates. After 4 days for RSV and 5 days for hMPV, the cells were fixed with 10% neutral buffered formalin. Cell monolayers were next blocked with block buffer comprising of 2% nonfat milk supplemented with 2% goat serum in PBS-T for 1 h. Next, the plates were washed three times with water, and 200 μL of MPV364 (for hMPV) or 101F (for RSV) (44) was added to a final concentration of 1 μg/mL (1:1,000 dilution) in blocking solution. The plates were then washed three times with water, and 200 μL of goat anti-human IgG HRP secondary antibody (Southern Biotech) diluted to a ratio of 1:2,000 in block buffer was added and incubated for 1 h at room temperature followed by 1 hr of incubation. Plates were washed again with water five times, and 200 μL of TrueBlue peroxidase substrate (SeraCare) was added to each well. The plates were incubated for 20–30 min until the plaques were clearly visible. Plaques were counted manually under a microscope. For influenza virus, lungs were collected and homogenized on day 7 post infection and viral titers were determined via plaque assay. Briefly, IAV challenged lung homogenates were serially diluted and plated on MDCK cells (2x overlay media MEM+2 μg/mL TPCK-treated trypsin+40mM HEPES+4mM L-glutamine+0.15% NaHCO_3_ and 1.4% Avicel) in 12-well plates. After 3 days, cells were fixed with Acetone : Methanol (20:80) and then stained with crystal violet. Plaques were then counted, and titers were determined from the average of three replicates.

### Bacterial burden studies

To determine bacterial burden in the lungs and blood of coinfected animals, the protocols described above were used. For IAV/*Spn* co-infection, groups of mice were euthanized at 12-, 24- and 36-hours post-infection mice, and blood and lungs were collected. For RSV/Spn and hMPV/Spn co-infections, groups of mice were euthanized on days 1, 2, and 3 post bacterial infection, and blood and lungs were collected. Blood was collected via cardiac puncture and was serially diluted and plated on TSA 5% sheep blood agar plates to determine bacterial titers. Lungs were extracted and homogenized in 1 mL of PBS, homogenates were then serially diluted and plated to determine bacterial titers as above.

## Results

### Assessment of bacterial vs viral mAb efficacy against secondary pneumococcal infection

**Figure 1:**
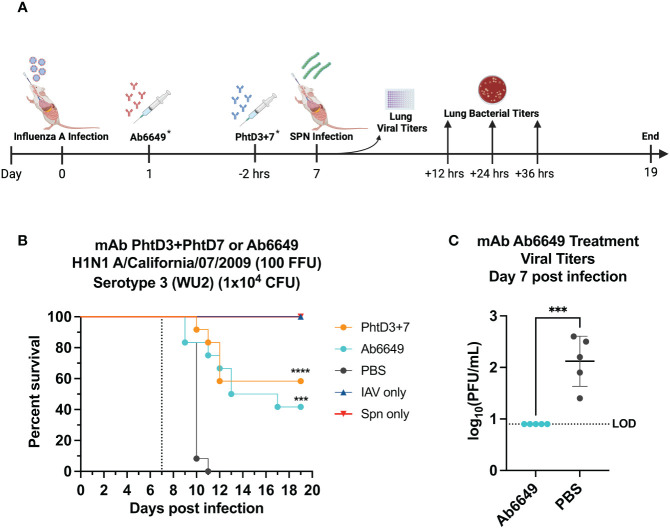
Evaluation of protective efficacy of PhtD3 + 7 cocktail and mAb Ab6649. **(A)** Timeline of IAV/*Spn* coinfection and data collection timepoints. *Each group of mice either received the Abb6649 mAb at Day 1 after IAV infection OR the PhtD3/7 mAb cocktail 2 hours prior to *Spn* infection. **(B)** Protective efficacy of mAb PhtD3 + 7 or Ab6649 in an IAV/*Spn* coinfection model. Mice were infected with H1N1 A/California/07/2009 at day 0 and with *Spn* serotype 3 *(*WU2) bacteria at day 7 (dotted line). Treatment with mAb Ab6649 occurred 24 hrs post IAV infection. Treatment with mAb PhtD3 + 7 occurred 2 hrs prior to pneumococcal infection. ***p = 0.0005, ****p < 0.0001 via log-rank (Mantel Cox) test compared with the PBS control group. n = 12 mice/group; IAV- and *Spn* infected groups had n = 5 mice/group. **(C)** Viral burden in the lungs after mAb Ab6649 treatment. Viral burden was assessed on 7 d after IAV infection. Treatment with mAb Ab6649 occurred 24 hrs post IAV infection. ***p = 0.0005 via unpaired t test. IAV, Influenza A Virus; Spn, S. pneumoniae; LOD, Limit of detection.

**Figure 2:**
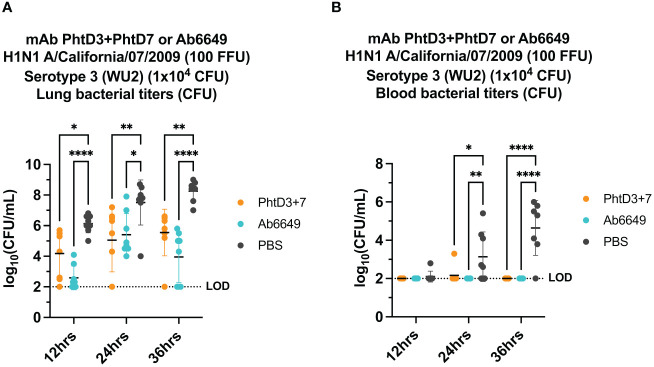
Bacterial burden in blood and lungs of mice in an Influenza virus/*Spn* coinfection model. **(A)** mAb PhtD3 + 7 effectively reduce bacterial blood titers at 12 hrs, 24 hrs and 36 hrs post *Spn* infection. Bacterial burden was assessed at 12 hrs, 24 hrs and 36 hrs post *Spn* infection. n = 8 mice/group. *p<0.05, **p<0.01, ****p<0.0001 by two-way ANOVA, compared to the PBS control group. **(B)** mAb PhtD3 + 7 effectively reduce bacterial lung titers at 12 hrs, 24 hrs and 36 hrs post *Spn* infection. Bacterial burden was assessed at 12 hrs, 24 hrs and 36 hrs post Spn infection. n = 8 mice/group. *p<0.05, **p<0.01, ****p<0.0001 by two-way ANOVA, compared to the PBS control group. LOD, Limit of detection.

In our previous studies, we isolated protective anti-PhtD mAbs and elucidated the mechanism of protection for mAb PhtD3 (41). Through immune depletion studies, we found that protection by mAb PhtD3 is likely mediated by macrophages and the complement system (41). While single anti-PhtD mAb treatment did not confer robust protection in an IAV/*Spn* murine coinfection model, a cocktail of mAbs PhtD3 and PhtD7 (PhtD3+PhtD7) did provide protection (41). Prior influenza infection in mice dysregulates the immune system impairing the antibacterial function of alveolar macrophages in C57BL/6 mice and depleting alveolar macrophages in BALB/c mice (19, 45). Other studies have shown that virus mediated damage and interactions of influenza virus and *Spn* also enhance the secondary bacterial infection (25, 46, 47). Due to virus mediated effects, we determined if mAbs targeting the virus would also confer protection upon secondary bacterial infection as the bacterial dose on its own is not lethal. Utilizing our previously established IAV/*Spn* coinfection model in C57BL/6 mice, we compared the protective efficacy of mAb Ab6649 to our mAb cocktail of PhtD3+PhtD7, which was previously shown to be protective against IAV/*Spn* coinfection using the H1N1 A/California/07/2009 (42) virus, as outlined in **Figure 1A**. In our first experiment, we administered 10 mg/kg of mAb Ab6649, which is a 3x higher dose than a protective dose previously shown (42), 24 hrs post IAV infection to one group, and 7.5 mg/kg each of mAb cocktail PhtD3+PhtD7 to a different group 2 hrs prior to *Spn* infection on day 7 post IAV infection. Compared to the PBS control group (0%), the mAb PhtD3 + 7 and mAb Ab6649 treated groups had a significant increase in survival (58 and 41%, respectively) with no significant difference between the different mAb treatment groups (**Figure 1B**). We also collected lung homogenates of IAV infected, Ab6649 treated animals and found that the PBS control animals had residual levels of IAV still present (average 2.1 log_10_ PFU/mL) whereas the Ab6649 treated animals had no detectable virus present in the lungs (**Figure 1C**). We next examined the kinetics and dissemination of bacterial growth after secondary pneumococcal infection in the IAV/*Spn* model. Utilizing the same infection and treatment plans as used in the survival study above, we collected the lungs and blood of mice at 12, 24 and 36 hrs post pneumococcal infection. At the first time point 12 hrs post *Spn* infection, we observed significantly reduced bacterial titers in the lungs of mAb PhtD3 + 7 and mAb Ab6649 treated mice compared to the PBS treated control group (average 4.18 versus 2.58 versus 6 log_10_ CFU/mL, respectively) (**Figure 2A**). To determine dissemination of the bacteria from the airways to the blood stream, we analyzed blood collected via cardiac puncture. At the 12 hrs time point, only one PBS control mouse had detectable levels of *Spn* in the bloodstream (**Figure 2B**). After 24 hrs, we saw an increase in lung bacterial titers of both mAb PhtD3 + 7 and mAb Ab6649 treated animals from the 12 hr timepoint (average 5.05 versus 5.41 log_10_ CFU/mL, respectively), however both groups were significantly lower than PBS control animals (average 7.5 log_10_ CFU/mL) (**Figure 2A**). At this same time point, we observed a significant increase in blood bacterial titers in the PBS control animals compared to mAb PhtD3 + 7 and Ab6649 treated animals (average 3.13 versus 2.16 versus 2 log_10_ CFU/mL, respectively) (**Figure 2B**). Finally at 36 hrs, we continued to see a significant decrease in lung bacterial titers in mAb PhtD3 + 7 and mAb Ab6649 treated animals compared to PBS treated animals (average 5.55 versus 3.95 versus 8.25 log_10_ CFU/mL, respectively) (**Figure 2A**). In parallel, we saw a significant decrease in blood bacterial titers in the mAb PhtD3 + 7 and mAb Ab6649 control animals compared PBS to treated animals (average 2 versus 2 versus 4.64 log_10_ CFU/mL, respectively) (**Figure 2B**). In conclusion, our experiments have determined that both mAb PhtD3 + 7 and mAb Ab6649 exhibit protective efficacy and reduce blood and lung titers in an IAV/*Spn* coinfection model.

### PhtD3 + 7 mAb cocktail protects mice against hMPV/Spn coinfection

**Figure 3:**
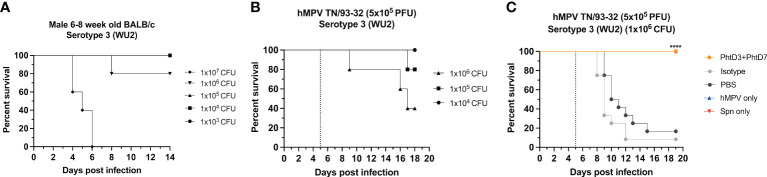
Coinfection with sublethal dose of hMPV followed by *Spn* causes mortality in mice. **(A)** Survival of BALB/c mice infected with decreasing doses of *Spn* serotype 3 (WU2). n = 5 mice/group. **(B)** Survival of BALB/c mice coinfected with hMPV TN/93-32 at day 0 and *Spn* serotype 3 (WU2) with decreasing doses at day 5 (dotted line) in 6- to 8-wk-old male mice. n = 5 mice/group. **(C)** Protective efficacy of mAb PhtD3 + 7 in a hMPV/*Spn* coinfection model. Mice were infected with hMPV TN/93-32 at day 0 and *Spn* serotype 3 (WU2) bacteria at day 5 (dotted line). Treatment with mAb PhtD3 + 7 occurred 2 hrs prior to *Spn* infection. ****p < 0.0001 via log-rank (Mantel Cox) test compared with the isotype control mAb group. n =12 mice/group.

**Figure 4:**
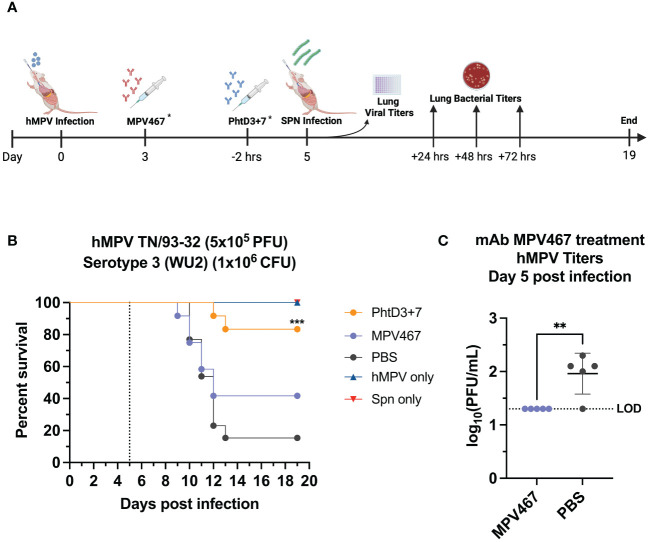
Coinfection with sublethal hMPV followed by *Spn* causes mortality in mice and is improved by PhtD3 + 7 mAb treatment. **(A)** Timeline of hMPV/*Spn* coinfection and data collection timepoints. *Each group of mice either received the MPV467 mAb at day 3 after hMPV infection OR the PhtD3/7 mAb cocktail 2 hours prior to *Spn infection*. **(B)** Protective efficacy of PhtD3 + 7 or MPV467 mAbs in a hMPV/*Spn* coinfection model. Mice were infected with hMPV TN/93-32 at day 0 and *Spn* serotype 3 (WU2) bacteria at day 5 (dotted line). Treatment with MPV467 occurred 72 hrs post hMPV infection. Treatment with mAb PhtD3 + 7 occurred 2 hrs prior to *Spn* infection. ***p < 0.0005 via log-rank (Mantel Cox) test compared with the PBS control group. Coinfected groups had n =12 mice/group; hMPV- and *Spn* infected groups had n = 5 mice/group. **(C)** Viral burden was assessed on day 5 after hMPV infection. Treatment with mAb MPV467 occurred day 3 post hMPV infection. **p < 0.01 via unpaired t test. LOD, Limit of detection.

**Figure 5:**
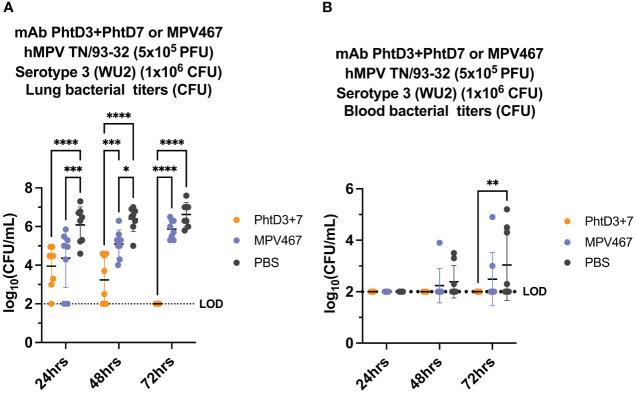
Bacterial burden in blood and lungs of hMPV/*Spn* coinfected mice. **(A)** mAb PhtD3 + 7 effectively reduce bacterial blood titers at 12 hrs, 24 hrs and 36 hrs post *Spn* infection. Bacterial burden was assessed at 24 hrs, 48 hrs and 72 hrs post *Spn* infection. n = 8 mice/group. **(B)** mAb PhtD3 + 7 effectively reduce bacterial lung titers at 24 hrs, 48 hrs and 72 hrs post *Spn* infection. Bacterial burden was assessed at 24 hrs, 48 hrs and 72 hrs post *Spn* infection. n = 8 mice/group. *p<0.05, **p<0.01, ***p<0.001, ****p<0.0001 by two-way ANOVA, compared to the PBS control group. LOD, Limit of detection.

Primary hMPV infection can also lead to secondary pneumococcal infection resulting in increased morbidity and mortality (7). hMPV-infected mice showed impaired recruitment of airway neutrophils, possibly leading to delayed bacterial clearance and exacerbated pulmonary inflammation after secondary infection with *Spn* (48). Since hMPV does not replicate well in C57BL/6 mice, we used BALB/c mice for the hMPV/*Spn* studies, as these mice are more susceptible to hMPV infection (49). Before testing the mAb PhtD3 + 7 cocktail, we established a hMPV/*Spn* coinfection model similar to a previous study (48). While BALB/c mice are more susceptible to hMPV infection, they are more resistant to *Spn* infection, so first we established a new dosing standard to determine the optimal dose of *Spn*. We infected 6-8 week old male BALB/c mice with 5 different doses, 10^7^, 10^6^, 10^5^, 10^4^ and 10^3^ CFUs of serotype 3 WU2. Mice infected with 10^5^, 10^4^ and 10^3^ had 100% survival and 10^6^ and 10^7^ had 80% and 0% survival, respectively (**Figure 3A**). Our previous studies have demonstrated that hMPV infection does not cause substantial disease in BALB/c mice using 5x10^5^ PFU of hMPV strain TN/93-32 (49). We first infected mice intranasally with 5x10^5^ PFU of hMPV TN/93-32, and 5 days later mice were infected with 3 different doses of *Spn at* 10^6^, 10^5^ and 10^4^ CFU/mouse. At 10^4^, 10^5^, and 10^6^ CFU/mouse we observed 0%, 20% and 60% mortality, respectively (**Figure 3B**). Based on these data, we utilized 10^6^ CFUs of *Spn* for subsequent studies due to the limited mortality seen in our singly infected animals. We then tested the mAb PhtD3 + 7 cocktail in the newly established coinfection model. On day 5 post hMPV infection, we administered 7.5 mg/kg each of mAb PhtD3 and mAb PhtD7 2 hrs prior to *Spn* infection. mAb PhtD3 + 7 showed a significant protective effect when compared to the isotype mAb and PBS control groups (100% versus 8.3% versus 16.6%, respectively) (**Figure 3C**). We next compared the protective effects of the PhtD3 + 7 mAb cocktail to a protective hMPV antibody, MPV467 (43). We observed 100% survival in both of the singly infected controls, Spn and hMPV only (**Figure 3C**). We utilized the previously published mAb MPV467, which targets the F protein of the virus and has been shown to neutralize hMPV *in vivo* (43). We followed the same experimental timeline as above, but added a group that was treated with MPV467 (10 mg/kg (43)) on day 3 post hMPV infection, as outlined in **Figure 4A**. mAb PhtD3 + 7 treatment was found to be the most protective compared to MPV467 treatment and the PBS control (83.3% versus 41.6% versus 15.3%, respectively) (**Figure 4B**). On day 5 post infection, we measured the lung viral titers of MPV467 treated mice compared to PBS treated animals and found a significant decrease with no detectable virus in MPV467 treated animals (average 1.3 versus 1.96 log_10_ PFU/mL, respectively) (**Figure 4C**). To better understand the dynamics of bacterial dissemination, we measured lung and blood bacterial titers at 24 hrs, 48 hrs and 72 hrs post *Spn* infection. At 24 hrs, we saw a significant reduction in lung bacterial titers in both the mAb PhtD3 + 7 and mAb MPV467 treated groups compared to PBS treated groups (average 3.95 versus 4.3 versus 6.08 log_10_ CFU/mL, respectively) (**Figure 5A**). Additionally, we found no detectable bacteria in the blood of mice (**Figure 5B**). At 48 hrs, we observed a significant reduction in lung bacterial titers in both the mAb PhtD3 + 7 and mAb MPV467 groups compared to PBS treated groups (average 3.2 versus 5 versus 6.3 log_10_ CFU/mL, respectively) (**Figure 5A**). At 48 hrs we began to see a rise in blood bacterial titers; however, mice treated with mAb PhtD3 + 7 still showed no detectable bacteria in the blood with MPV 467 and PBS treated showing a slight increase in titers (average 2 versus 2.23 versus 2.38 log_10_ CFU/mL, respectively) (**Figure 5B**). Finally, at 72 hrs post *Spn* infection we saw no detectable bacteria in the lungs of mAb PhtD3 + 7 treated animals; however, we saw a further increase in titers of both MPV467 treated and PBS treated groups (average 2 versus 5.7 versus 6.6 log_10_ CFU/mL, respectively) (**Figure 5A**). Continuing the trend from the lungs, we detected no bacteria in the blood of PhtD3 + 7 animals but an increase in titers for MPV467 and PBS (average 2 versus 2.48 versus 3.03 log_10_ CFU/mL, respectively) (**Figure 5B**). In conclusion, the hMPV/*Spn* coinfection model revealed that the PhtD3 + 7 mAb cocktail substantially enhanced survival rates and decreased lung and blood bacterial levels, while mAb MPV467 provided moderate protection.

### PhtD3 + 7 mAb cocktail protects mice against RSV/Spn coinfection

**Figure 6:**
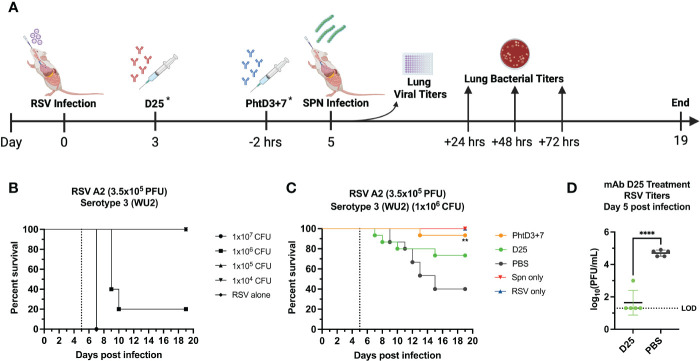
Coinfection with sublethal RSV followed by *Spn* causes mortality in mice. **(A)** Timeline of RSV/*Spn* coinfection and data collection timepoints. *Each group of mice either received the D25 mAb at Day 3 after RSV infection OR the PhtD3/7 mAb cocktail 2 hours prior to *Spn* infection. **(B)** Survival of BALB/c mice coinfected with RSV A2 at day 0 and *Spn* serotype 3 (strain WU2) at day 5 (dotted line) in 6- to 8-wk-old male mice. n = 5 mice/group. **(C)** Protective efficacy of PhtD3 + 7 or D25 mAbs in an RSV/*Spn* coinfection model. Mice were infected with RSV A2 at day 0 and with *Spn* serotype 3 (strain WU2) bacteria at day 5 (dotted line). Treatment with D25 occurred 72 hrs post RSV infection. Treatment with mAb PhtD3 + 7 occurred 2 hrs prior to *Spn* serotype 3 (strain WU2) infection. **p < 0.01 via log-rank (Mantel Cox) test compared with the PBS control group. Coinfected groups had n =12 mice/group; RSV- and *Spn* infected groups had n = 5 mice/group. **(D)** Viral burden was assessed on day 5 after RSV A2 infection. Treatment with mAb D25 occurred 3 d post RSV infection. ****p < 0.0001 via unpaired t test. LOD, Limit of detection.

**Figure 7:**
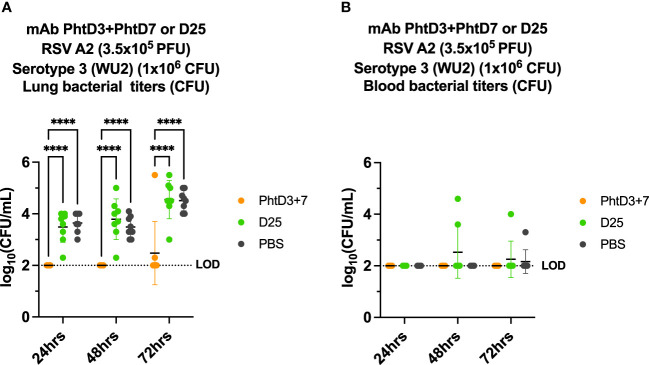
Bacterial burden in blood and lungs of RSV/*Spn* coinfected mice. **(A)** mAb PhtD3 + 7 effectively reduce bacterial blood titers at 12 hrs, 24 hrs and 36 hrs post *Spn* infection. Bacterial burden was assessed at 24 hrs, 48 hrs and 72 hrs post *Spn* infection. n = 8 mice/group. **(B)** mAb PhtD3 + 7 effectively reduce bacterial lung titers at 24 hrs, 48 hrs and 72 hrs post *Spn* infection. Bacterial burden was assessed at 24 hrs, 48 hrs and 72 hrs post *Spn* infection. n = 8 mice/group. ****p<0.0001 by two-way ANOVA, compared to the PBS control group. LOD, Limit of detection.

Primary RSV infection has also been shown to lead to an increased incidence and severity of secondary pneumococcal infection (2, 6, 50). Similar to hMPV, we established a coinfection model in BALB/c mice to allow for sufficient replication of the RSV in the lungs of the mice, as outlined in **Figure 6A**. Our previous studies have demonstrated that RSV A2 does not cause disease at a dose of 3.5x10^5^ PFU/mouse (44). We first infected mice intranasally with 3.5x10^5^ PFU of RSV A2, and 5 days later we infected groups of mice with 4 different doses of *Spn*, 10^7^, 10^6^, 10^5^ and 10^4^ CFU/mouse. At 10^4^, 10^5^, 10^6^, 10^7^ CFU/mouse we observed 0%, 0%, 80% and 100% mortality, respectively (**Figure 6B**). Comparable to the hMPV/*Spn* infection, we used a 10^6^ dose *Spn* in the RSV/*Spn* model. We utilized mAb D25, which had previously been shown to target the F protein of RSV and was neutralizing *in vivo* (51). mAb D25 is the precursor to the recently approved RSV prophylactic mAb nirsevimab (51). We first performed a survival study to compare mAb PhtD3 + 7 to mAb D25 treatment. We observed a significant protective effect in both mAb PhtD3 + 7 and mAb D25 treated mice compared to PBS treated control (93.3% versus 73.3% versus 40%, respectively) (**Figure 6C**). Additionally, our singly infected animals showed 100% survival as expected. We then measured lung viral titers on day 5 post RSV infection. Mice administered mAb D25 on day 3 post RSV infection had a significant decrease in detectable virus titers compared to PBS (average 1.64 versus 4.7 log_10_ PFU/mL, respectively) (**Figure 6D**). We next measured lung and blood bacterial titers at 24 hrs, 48 hrs and 72 hrs post *Spn* infection. At 24 hrs, we saw a significant reduction in lung bacterial titers in both the mAb PhtD3 + 7 and mAb D25 treated groups compared to the PBS treated group (average 2 versus 3.48 versus 3.64 log_10_ CFU/mL, respectively) with no bacteria detected in the PhtD3 + 7 group (**Figure 7A**). Additionally, we found no detectable bacteria in the blood of any groups (**Figure 7B**). At 48 hrs, we observed a significant reduction in lung bacterial titers in the mAb PhtD3 + 7 treated group, with no detectable bacteria compared to the mAb D25 and PBS treated groups (average 2 versus 3.78 versus 3.48 log_10_ CFU/mL, respectively) (**Figure 7A**). We did detect bacteria in the blood of two animals in the mAb D25 treated group (**Figure 7B**). Lastly, at 72 hrs post bacterial infection, we observed only two animals with bacteria in the lungs in the mAb PhtD3 + 7 treated animals; however, we observed a further increase in bacterial titers in both mAb D25 and PBS treated groups (average 2.47 versus 4.55 versus 4.51 log_10_ CFU/mL, respectively) (**Figure 7A**). Furthermore, we detected no bacteria in the blood of mAb PhtD3 + 7 treated animals, but one animal in each mAb D25 and PBS treated groups had detectable bacteria (**Figure 7B**). In conclusion, the RSV/*Spn* coinfection study demonstrated the exceptional protective qualities and effectiveness of PhtD3 + 7 in significantly reducing lung and blood bacterial titers, and while mAb D25 treatment did improve survival, it failed to exhibit a similar reduction in bacterial titers.

## Discussion

Taken together, this study highlights the potent protective capacity of the anti-pneumococcal human mAb cocktail, PhtD3+PhtD7, in combating coinfections involving IAV/*Spn*, hMPV/*Spn*, and RSV/*Spn*. The PhtD3 + 7 mAb cocktail outperformed individual human mAbs targeting each specific virus. Moreover, the PhtD3 + 7 mAb cocktail reduced lung bacterial titers and prevented bacterial dissemination into the bloodstream, surpassing the effectiveness of our antiviral mAbs. Building upon our past investigations (41, 52), this study reaffirms the protective capabilities of anti-PhtD mAbs.

Primary influenza virus infection has been shown to predispose hosts to synergistic secondary bacterial infections leading to increased morbidity and mortality (45, 53). Influenza virus achieves this through a variety of mechanisms. The airways can become compromised leading to a decrease in mucocilliary clearance, destruction of tight junctions, increase in available nutrients for the bacteria, and exposure of host receptors that *Spn* can utilize to bind to host cells (12, 24, 45, 54). In addition to these physical phenomena, immune dysregulation occurs on multiple fronts. The dysregulation of alveolar macrophages is an important aspect as macrophages are essential in host defense against *Spn* (45). Neutrophils are also susceptible to viral priming, further impairing the innate immune response to secondary bacterial infection (55). This immune dysfunction is driven by IFN- γ and its down streaming signaling impairing phagocytic cells, leading to a more inflammatory state (17, 22, 56). Based on the body of literature and our previous study (41) demonstrating the protective efficacy our PhtD mAbs in an IAV/*Spn* coinfection model, we wanted to determine if targeting the virus prior to infection with *Spn* would also be protective. Utilizing the previously described mAb Ab6649 (42) 24 hrs post viral infection, we found similar levels of protection between Ab6649 and the PhtD3 + 7 mAb cocktail. Upon titering of mouse lungs at day 7, we found that treatment with Ab6649 led to no detectable virus in the lungs at the point of challenge with *Spn*, but low levels of virus were present in the PBS control mice. To further dive into the kinetics of infection, we measured lung and blood titers at 12, 24 and 36 hrs post *Spn* infection. Both antibodies significantly reduced bacterial lung and blood titers compared to our PBS control. our results suggest that our monoclonal antibodies (mAbs) effectively halted the bacteria’s invasion into the bloodstream, within the time points we’ve evaluated. Resolution of the viral infection by day 7 did show positive protective effects; however, both approaches targeting the virus or the bacteria yielded positive clinical outcomes, but seemingly through different mechanisms as they have different targets. It is likely that the Ab6649 treatment prevented widespread damage to the epithelium by neutralizing the virus and preventing an excessive inflammatory response, thereby allowing the innate immune system to keep *Spn* under control. The ability of PhtD3 + 7 to remain protective in a dysregulated immune state. As mentioned before, primary viral infections dysregulate macrophages antimicrobial abilities (19, 45), and we have shown mAb PhtD3 requires macrophages to be protective (41). Further studies are needed to decipher the exact protective mechanism in the context of IAV/*Spn* coinfections.

hMPV and RSV, both members of the *Pneumoviridae* family, are two major viral causes of secondary bacterial coinfections; however, these viral infections cannot be appropriately modeled using C57BL/6 mice. We established a model in BALB/c mice to enable viral replication, despite their enhanced resistance to *Spn* infection. It has been shown that C57BL/6 mice have more efficient Alveolar macrophage mediated bacterial clearance (57), whereas BALB/c mice have a more robust neutrophil response (58). C57BL/6 also demonstrate a greater inflammatory response compared to BALB/c mice (59). We found that for both coinfections, day 5 post viral infection was the optimal time to infect with *Spn*. It has been shown that a preceding hMPV infection makes the host more susceptible to *Spn* adherence (48). In RSV/*Spn* coinfections, studies show that the lethal synergy occurs via the formation of RSV-*Spn* complexes and expression of viral glycoproteins on the surface of infected cells, leading to increased attachment and adherence (60, 61). Due to these direct interactions between hMPV or RSV and *Spn*, we hypothesized that a mAb neutralizing these viruses would have therapeutic potential. Similar to our IAV coinfection, we showed that in PBS treated animals, there were low levels of virus present in the lungs, but upon antiviral mAb treatment, MPV467 (hMPV) and D25 (RSV), virus was not detectable in MPV467-treated animals. We saw significant protection with our PhtD3 + 7 mAb cocktail in both coinfection models and this protection was greater than our antiviral mAbs. Our data indicates that using antiviral mAbs to neutralize the virus prior to *Spn* infection is not as effective as using mAb PhtD3 + 7 for protection. We believe the lethal synergy between hMPV or RSV and *Spn* stems from immune dysfunction, not direct interactions between the virus and bacteria. Due to the less severe nature of these coinfections, we extended the time points for measuring bacterial titers up to 72 hrs post *Spn* infection. In the hMPV/*Spn* coinfection model, we saw a decrease in lung bacterial titers in our PhtD3 + 7 group with no bacteria detected at 72 hrs post *Spn* infection. Interestingly, in our RSV coinfection model, while D25 treatment did increase survival, there was no decrease in lung titers compared to our PBS control, whereas PhtD3 + 7 mAb cocktail effectively decreased lung titers below the limit of detection. It is possible that D25 treatment prevents robust immune dysfunction, which would explain the protective effect seen, but the lack of reduction in bacterial titers. Once again mAb PhtD3 + 7 effectively prevented detectable levels of bacteria from entering the bloodstream, which likely points to a key protective feature.

Prior research has highlighted the significance of physical damage and immune dysfunction resulting from primary viral infections, which can lead to subsequent bacterial infections (54). While we have previously pinpointed the protective role of PhtD3 *in vivo*, driven by macrophages, the protective mechanism of mAb PhtD7 still requires further investigation. Our ongoing research aims to solidify the notion that targeting conserved surface antigens is a fruitful approach not only for individual infections, but also for co-infections. To our knowledge, this is the first study to demonstrate mAbs to be effective across three distinct co-infection models. Future studies will delve deeper into how PhtD3 + 7 overcomes phagocytic deficiencies and will ultimately determine if a combination of antiviral and antibacterial monoclonal antibodies offers the most promising therapeutic solution for viral/bacterial co-infections.

## Data availability statement

The raw data supporting the conclusions of this article will be made available by the authors, without undue reservation.

## Ethics statement

The animal study was approved by University of Georgia Institutional Animal Care and Use Committee. The study was conducted in accordance with the local legislation and institutional requirements.

## Author contributions

AG: Conceptualization, Formal analysis, Investigation, Methodology, Writing – original draft, Writing – review & editing. LM: Formal analysis, Investigation, Methodology, Writing – review & editing. AM: Formal analysis, Investigation, Writing – review & editing. RM: Investigation, Writing – review & editing. JM: Conceptualization, Funding acquisition, Supervision, Writing – original draft, Writing – review & editing.

## References

[1] Weiser JN Ferreira DM Paton JC. Streptococcus pneumoniae: transmission, colonization and invasion. Nat Rev Microbiol. (2018) 16:355–67. doi: 10.1038/s41579-018-0001-8 PMC5949087 29599457

[2] Perez A Lively JY Curns A Weinberg GA Halasa NB Mary. Morbidity and Mortality Weekly Report Respiratory Virus Surveillance Among Children with Acute Respiratory Illnesses-New Vaccine Surveillance Network, United States, 2016-2021 (2022). Available online at: https://www.cdc.gov/mmwr/mmwr_continuingEducation.html. 10.15585/mmwr.mm7140a1 PMC9541034 36201373

[3] McCullers JA. The co-pathogenesis of influenza viruses with bacteria in the lung. Nat Rev Microbiol. (2014) 12:252–62. doi: 10.1038/nrmicro3231 24590244

[4] Morens DM Taubenberger JK Fauci AS. Predominant role of bacterial pneumonia as a cause of death in pandemic influenza: implications for pandemic influenza preparedness. J Infect Dis. (2008) 198:962–70. doi: 10.1086/591708 PMC2599911 18710327

[5] Chi H Huang Y-C Liu C-C Chang K-Y Huang Y-C Lin H-C. Characteristics and etiology of hospitalized pediatric community-acquired pneumonia in Taiwan. J Formosan Med Assoc. (2020) 119:1490–9. doi: 10.1016/j.jfma.2020.07.014 PMC7363436 32682702

[6] Liu Y Ling L Wong SH Wang MHT Fitzgerald JR Zou X. Outcomes of respiratory viral-bacterial co-infection in adult hospitalized patients. EClinicalMedicine. (2021) 37:100955. doi: 10.1016/j.eclinm.2021.100955 34386745 PMC8343259

[7] Madhi SA Ludewick H Kuwanda L van Niekerk N Cutland C Little T. Pneumococcal coinfection with human metapneumovirus. J Infect Dis. (2006) 193:1236–43. doi: 10.1086/503053 16586360

[8] Vidaur L Totorika I Montes M Vicente D Rello J Cilla G. Human metapneumovirus as cause of severe community-acquired pneumonia in adults: insights from a ten-year molecular and epidemiological analysis. Ann Intensive Care. (2019) 9:86. doi: 10.1186/s13613-019-0559-y 31342206 PMC6656825

[9] Monsalvo AC Batalle JP Lopez MF Krause JC Klemenc J Hernandez JZ. Severe pandemic 2009 H1N1 influenza disease due to pathogenic immune complexes. Nat Med. (2011) 17:195–9. doi: 10.1038/nm.2262 PMC3034774 21131958

[10] Klein EY Monteforte B Gupta A Jiang W May L Hsieh YH. The frequency of influenza and bacterial coinfection: a systematic review and meta-analysis. Influenza other Respir Viruses. (2016) 10:394–403. doi: 10.1111/irv.12398 27232677 PMC4947938

[11] Godefroy R Giraud-gatineau A Jimeno M-T Edouard S Meddeb L Zandotti C. Respiratory syncytial virus infection: its propensity for bacterial coinfection and related mortality in elderly adults. Open Forum Infect Dis. (2020) 7:ofaa546. doi: 10.1093/ofid/ofaa546 33335940 PMC7733236

[12] Pittet LA Hall-Stoodley L Rutkowski MR Harmsen AG. Influenza virus infection decreases tracheal mucociliary velocity and clearance of Streptococcus pneumoniae. Am J Respir Cell Mol Biol. (2010) 42:450–60. doi: 10.1165/rcmb.2007-0417OC PMC2848738 19520922

[13] Karwelat D Schmeck B Ringel M Benedikter BJ Hübner K Beinborn I. Influenza virus-mediated suppression of bronchial Chitinase-3-like 1 secretion promotes secondary pneumococcal infection. FASEB J. (2020) 34:16432–48. doi: 10.1096/fj.201902988RR 33095949

[14] Ellis GT Davidson S Crotta S Branzk N Papayannopoulos V Wack A. TRAIL+ monocytes and monocyte-related cells cause lung damage and thereby increase susceptibility to influenza–S treptococcus pneumoniae coinfection. EMBO Rep. (2015) 16:1203–18. doi: 10.15252/embr.201540473 PMC4576987 26265006

[15] van der Sluijs KF van Elden LJR Nijhuis M Schuurman R Florquin S Shimizu T. Involvement of the platelet-activating factor receptor in host defense against Streptococcus pneumoniae during postinfluenza pneumonia. Am J physiology-lung Cell Mol Physiol. (2006) 290:L194–9. doi: 10.1152/ajplung.00050.2005 16100290

[16] Dela Cruz CS Liu W He CH Jacoby A Gornitzky A Ma B. Chitinase 3-like-1 promotes Streptococcus pneumoniae killing and augments host tolerance to lung antibacterial responses. Cell Host Microbe. (2012) 12:34–46. doi: 10.1016/j.chom.2012.05.017 22817986 PMC3613130

[17] Sun K Metzger DW. Inhibition of pulmonary antibacterial defense by interferon-γ during recovery from influenza infection. Nat Med. (2008) 14:558–64. doi: 10.1038/nm1765 18438414

[18] Gou X Yuan J Wang H Wang X Xiao J Chen J. IL-6 during influenza-streptococcus pneumoniae co-infected pneumonia—a protector. Front Immunol. (2020) 10:3102. doi: 10.3389/fimmu.2019.03102 32038632 PMC6985362

[19] Verma AK Bansal S Bauer C Muralidharan A Sun K. Influenza infection induces alveolar macrophage dysfunction and thereby enables noninvasive Streptococcus pneumoniae to cause deadly pneumonia. J Immunol. (2020) 205:1601–7. doi: 10.4049/jimmunol.2000094 PMC7484308 32796026

[20] Wu M Gibbons JG DeLoid GM Bedugnis AS Thimmulappa RK Biswal S. Immunomodulators targeting MARCO expression improve resistance to postinfluenza bacterial pneumonia. Am J Physiology-Lung Cell Mol Physiol. (2017) 313:L138–53. doi: 10.1152/ajplung.00075.2017 PMC5538876 28408365

[21] Didierlaurent A Goulding J Patel S Snelgrove R Low L Bebien M. Sustained desensitization to bacterial Toll-like receptor ligands after resolutionof respiratory influenza infection. J Exp Med. (2008) 205:323–9. doi: 10.1084/jem.20070891 PMC2271005 18227219

[22] Nakamura S Davis KM Weiser JN. Synergistic stimulation of type I interferons during influenza virus coinfection promotes Streptococcus pneumoniae colonization in mice. J Clin Invest. (2011) 121. doi: 10.1172/JCI57762 PMC3163966 21841308

[23] Cao J Wang D Xu F Gong Y Wang H Song Z. Activation of IL-27 signalling promotes development of postinfluenza pneumococcal pneumonia. EMBO Mol Med. (2014) 6:120–40. doi: 10.1002/emmm.201302890 PMC3936494 24408967

[24] Sender V Hentrich K Pathak A Tan Qian Ler A Embaie BT Lundstrom SL. Capillary leakage provides nutrients and antioxidants for rapid pneumococcal proliferation in influenza-infected lower airways. Proc Natl Acad Sci. (2020) 117:31386–97. doi: 10.1073/pnas.2012265117 PMC7733805 33229573

[25] Siegel SJ Roche AM Weiser JN. Influenza promotes pneumococcal growth during coinfection by providing host sialylated substrates as a nutrient source. Cell Host Microbe. (2014) 16:55–67. doi: 10.1016/j.chom.2014.06.005 25011108 PMC4096718

[26] Pettigrew MM Marks LR Kong Y Gent JF Roche-Hakansson H Hakansson AP. Dynamic changes in the Streptococcus pneumoniae transcriptome during transition from biofilm formation to invasive disease upon influenza A virus infection. Infect Immun. (2014) 82:4607–19. doi: 10.1128/IAI.02225-14 PMC4249342 25135685

[27] Akhter F Womack E Vidal JE Le Breton Y McIver KS Pawar S. Hemoglobin stimulates vigorous growth of Streptococcus pneumoniae and shapes the pathogen’s global transcriptome. Sci Rep. (2020) 10:15202. doi: 10.1038/s41598-020-71910-1 32938947 PMC7494912

[28] Brealey JC Young PR Sloots TP Ware RS Lambert SB Sly PD. Bacterial colonization dynamics associated with respiratory syncytial virus during early childhood. Pediatr Pulmonol. (2020) 55:1237–45. doi: 10.1002/ppul.24715 32176838

[29] He W-Q Gianacas C Muscatello DJ Newall AT McIntyre P Cheng AC. Effectiveness of influenza vaccination in reducing influenza-like illness and related antibiotic prescriptions in adults from a primary care-based case-control study. J Infect. (2022) 85:660–5. doi: 10.1016/j.jinf.2022.10.028 36288784

[30] Farrar JL Childs L Ouattara M Akhter F Britton A Pilishvili T. Systematic review and meta-analysis of the efficacy and effectiveness of pneumococcal vaccines in adults. Pathogens. (2023) 12. doi: 10.3390/pathogens12050732 PMC10222197 37242402

[31] Ngocho JS Magoma B Olomi GA Mahande MJ Msuya SE de Jonge MI. Effectiveness of pneumococcal conjugate vaccines against invasive pneumococcal disease among children under five years of age in Africa: A systematic review. PLoS One. (2019) 14:e0212295. doi: 10.1371/journal.pone.0212295 30779801 PMC6380553

[32] Mclean HQ Petrie JG Hanson KE Meece JK Rolfes MA Sylvester GC. Morbidity and Mortality Weekly Report Interim Estimates of 2022-23 Seasonal Influenza Vaccine Effectiveness-Wisconsin, October 2022-February 2023 (2023). Available online at: https://www.cdc.gov/mmwr/mmwr_continuingEducation.html. 10.15585/mmwr.mm7208a1 PMC9949852 36821715

[33] Hsiao A Hansen J Timbol J Lewis N Isturiz R Alexander-Parrish R. Incidence and estimated vaccine effectiveness against hospitalizations for all-cause pneumonia among older US adults who were vaccinated and not vaccinated with 13-valent pneumococcal conjugate vaccine. JAMA Netw Open. (2022) 5:e221111–e221111. doi: 10.1001/jamanetworkopen.2022.1111 35302634 PMC8933738

[34] Metzger DW Furuya Y Salmon SL Roberts S Sun K. Limited efficacy of antibacterial vaccination against secondary serotype 3 pneumococcal pneumonia following influenza infection. J Infect Dis. (2015) 212:445–52. doi: 10.1093/infdis/jiv066 PMC4615793 25649173

[35] Thors V Christensen H Morales-Aza B Vipond I Muir P Finn A. The effects of live attenuated influenza vaccine on nasopharyngeal bacteria in healthy 2 to 4 year olds. A randomized controlled trial. Am J Respir Crit Care Med. (2016) 193:1401–9. doi: 10.1164/rccm.201510-2000OC PMC4910891 26742001

[36] Jirru E Lee SF Harris R Yang JJ Cho SJ Stout-Delgado H. Impact of Influenza on Pneumococcal Vaccine Effectiveness during *Streptococcus pneumoniae* Infection in Aged Murine Lung. Vaccines (Basel). (2020) 8. doi: 10.3390/vaccines8020298 PMC7349919 32545261

[37] McCullers JA. Preventing and treating secondary bacterial infections with antiviral agents. Antivir Ther. (2011) 16:123–35. doi: 10.3851/IMP1730 PMC4907367 21447860

[38] Smith AM. Quantifying the therapeutic requirements and potential for combination therapy to prevent bacterial coinfection during influenza. J Pharmacokinet Pharmacodyn. (2017) 44:81–93. doi: 10.1007/s10928-016-9494-9 27679506 PMC5376398

[39] McCullers JA. Effect of antiviral treatment on the outcome of secondary bacterial pneumonia after influenza. J Infect Dis. (2004) 190:519–26. doi: 10.1086/421525 15243927

[40] English BK. Limitations of beta-lactam therapy for infections caused by susceptible Gram-positive bacteria. J Infect. (2014) 69:S5–9. doi: 10.1016/j.jinf.2014.07.010 25124369

[41] Gingerich AD Royer F McCormick AL Scasny A Vidal JE Mousa JJ. Synergistic protection against secondary pneumococcal infection by human monoclonal antibodies targeting distinct epitopes. J Immunol. (2023) 210:50–60. doi: 10.4049/jimmunol.2200349 36351696 PMC9898123

[42] Li T Chen J Zheng Q Xue W Zhang L Rong R. Identification of a cross-neutralizing antibody that targets the receptor binding site of H1N1 and H5N1 influenza viruses. Nat Commun. (2022) 13:5182. doi: 10.1038/s41467-022-32926-5 36056024 PMC9439264

[43] Banerjee A Huang J Rush SA Murray J Gingerich AD Royer F. Structural basis for ultrapotent antibody-mediated neutralization of human metapneumovirus. Proc Natl Acad Sci USA. (2022) 119:e2203326119. doi: 10.1073/pnas.2203326119 35696580 PMC9231621

[44] Huang J Miller RJ Mousa JJ. A Pan-Pneumovirus vaccine based on immunodominant epitopes of the fusion protein. Front Immunol. (2022) 13:941865. doi: 10.3389/fimmu.2022.941865 36003370 PMC9393700

[45] Ghoneim HE Thomas PG McCullers JA. Depletion of alveolar macrophages during influenza infection facilitates bacterial superinfections. J Immunol. (2013) 191:1250–9. doi: 10.4049/jimmunol.1300014 PMC4907362 23804714

[46] Mikušová M Tomčíková K Briestenská K Kostolanský F Varečková E. The contribution of viral proteins to the synergy of influenza and bacterial co-infection. Viruses. (2022) 14. doi: 10.3390/v14051064 PMC9143653 35632805

[47] Rowe HM Meliopoulos VA Iverson A Bomme P Schultz-Cherry S Rosch JW. Direct interactions with influenza promote bacterial adherence during respiratory infections. Nat Microbiol. (2019) 4:1328–36. doi: 10.1038/s41564-019-0447-0 PMC7069060 31110359

[48] Lai S-H Liao S-L Wong K-S Lin T-Y. Preceding human metapneumovirus infection increases adherence of Streptococcus pneumoniae and severity of murine pneumococcal pneumonia. J Microbiol Immunol Infect. (2016) 49:216–24. doi: 10.1016/j.jmii.2014.04.008 24931548

[49] Alvarez R Harrod KS Shieh W-J Zaki S Tripp RA. Human metapneumovirus persists in BALB/c mice despite the presence of neutralizing antibodies. J Virol. (2004) 78:14003–11. doi: 10.1128/JVI.78.24.14003-14011.2004 PMC533920 15564507

[50] Nguyen DT Louwen R Elberse K van Amerongen G Yüksel S Luijendijk A. Streptococcus pneumoniae enhances human respiratory syncytial virus infection *in vitro* and *in vivo* . PloS One. (2015) 10:e0127098–. doi: 10.1371/journal.pone.0127098 PMC4430531 25970287

[51] Zhu Q McLellan JS Kallewaard NL Ulbrandt ND Palaszynski S Zhang J. A highly potent extended half-life antibody as a potential RSV vaccine surrogate for all infants. Sci Transl Med. (2017) 9:eaaj1928. doi: 10.1126/scitranslmed.aaj1928 28469033

[52] Huang J Gingerich AD Royer F Paschall AV Pena-Briseno A Avci FY. Broadly reactive human monoclonal antibodies targeting the pneumococcal histidine triad protein protect against fatal pneumococcal infection. Infect Immun. (2021) 89:e00747–20. doi: 10.1128/IAI.00747-20 PMC8091081 33649050

[53] Metzger DW Sun K. Immune dysfunction and bacterial coinfections following influenza. J Immunol. (2013) 191:2047–52. doi: 10.4049/jimmunol.1301152 PMC3760235 23964104

[54] Sender V Hentrich K Henriques-Normark B. Virus-induced changes of the respiratory tract environment promote secondary infections with streptococcus pneumoniae. Front Cell Infect Microbiol. (2021) 11. doi: 10.3389/fcimb.2021.643326 PMC8019817 33828999

[55] McNamee LA Harmsen AG. Both influenza-induced neutrophil dysfunction and neutrophil-independent mechanisms contribute to increased susceptibility to a secondary Streptococcus pneumoniae infection. Infect Immun. (2006) 74:6707–21. doi: 10.1128/IAI.00789-06 PMC1698099 16982840

[56] Barman TK Racine R Bonin JL Califano D Salmon SL Metzger DW. Sequential targeting of interferon pathways for increased host resistance to bacterial superinfection during influenza. PLoS Pathog. (2021) 17:e1009405. doi: 10.1371/journal.ppat.1009405 33690728 PMC7978370

[57] Keer S Yan G M. DW. Analysis of murine genetic predisposition to pneumococcal infection reveals a critical role of alveolar macrophages in maintaining the sterility of the lower respiratory tract. Infect Immun. (2011) 79:1842–7. doi: 10.1128/IAI.01143-10 PMC3088159 21321074

[58] Gingles NA Alexander JE Kadioglu A Andrew PW Kerr A Mitchell TJ. Role of genetic resistance in invasive pneumococcal infection: identification and study of susceptibility and resistance in inbred mouse strains. Infect Immun. (2001) 69:426–34. doi: 10.1128/IAI.69.1.426-434.2001 PMC97900 11119534

[59] Preston JA Beagley KW Gibson PG Hansbro PM. Genetic background affects susceptibility in nonfatal pneumococcal bronchopneumonia. Eur Respir J. (2004) 23:224–31. doi: 10.1183/09031936.03.00081403 14979496

[60] Hament J-M Aerts PC Fleer A van Dijk H Harmsen T Kimpen JLL. Enhanced adherence of streptococcus pneumoniae to human epithelial cells infected with respiratory syncytial virus. Pediatr Res. (2004) 55:972–8. doi: 10.1203/01.PDR.0000127431.11750.D9 15103015

[61] Hament J-M Aerts PC Fleer A van Dijk H Harmsen T Kimpen JLL. Direct binding of respiratory syncytial virus to pneumococci: A phenomenon that enhances both pneumococcal adherence to human epithelial cells and pneumococcal invasiveness in a murine model. Pediatr Res. (2005) 58:1198–203. doi: 10.1203/01.pdr.0000188699.55279.1b 16306193

